# Change in Postprandial Level of Remnant Cholesterol After a Daily Breakfast in Chinese Patients With Hypertension

**DOI:** 10.3389/fcvm.2021.685385

**Published:** 2021-06-15

**Authors:** Jin Xu, Peiliu Qu, Xiao Du, Qunyan Xiang, Liling Guo, Liyuan Zhu, Yangrong Tan, Yan Fu, Tie Wen, Ling Liu

**Affiliations:** ^1^Department of Cardiovascular Medicine, The Second Xiangya Hospital, Central South University, Changsha, China; ^2^Research Institute of Blood Lipid and Atherosclerosis, Central South University, Changsha, China; ^3^Modern Cardiovascular Disease Clinical Technology Research Center of Hunan Province, Changsha, China; ^4^Cardiovascular Disease Research Center of Hunan Province, Changsha, China; ^5^Department of Emergency Medicine, Second Xiangya Hospital, Central South University, Changsha, China; ^6^Emergency Medicine and Difficult Diseases Institute, Second Xiangya Hospital, Central South University, Changsha, China

**Keywords:** hypertension, postprandial, remnant cholesterol, cut-off point, a daily breakfast

## Abstract

**Background:** Hypertension (HBP) is usually accompanied by hypertriglyceridemia that represents the increased triglyceride-rich lipoproteins and cholesterol content in remnant lipoproteins [i.e., remnant cholesterol (RC)]. According to the European Atherosclerosis Society (EAS), high RC (HRC) is defined as fasting RC ≥0.8 mmol/L and/or postprandial RC ≥0.9 mmol/L. However, little is known about postprandial change in RC level after a daily meal in Chinese patients with HBP.

**Methods:** One hundred thirty-five subjects, including 90 hypertensive patients (HBP group) and 45 non-HBP controls (CON group), were recruited in this study. Serum levels of blood lipids, including calculated RC, were explored at 0, 2, and 4 h after a daily breakfast. Receiver operating characteristic (ROC) curve analysis was used to determine the cutoff point of postprandial HRC.

**Results:** Fasting TG and RC levels were significantly higher in the HBP group (*P* < 0.05), both of which increased significantly after a daily meal in the two groups (*P* < 0.05). Moreover, postprandial RC level was significantly higher in the HBP group (*P* < 0.05). ROC curve analysis showed that the optimal cutoff point for RC after a daily meal to predict HRC corresponding to fasting RC of 0.8 mmol/L was 0.91 mmol/L, which was very close to that recommended by the EAS, i.e., 0.9 mmol/L. Fasting HRC was found in 31.1% of hypertensive patients but not in the controls. According to the postprandial cutoff point, postprandial HRC was found in approximately half of hypertensive patients and ~1-third of the controls.

**Conclusion:** Postprandial RC level increased significantly after a daily meal, and hypertensive patients had higher percentage of HRC at both fasting and postprandial states. More importantly, the detection of postprandial lipids could be helpful to find HRC.

## Background

As important atherogenic risk factors, hypertension (HBP) and hyperlipidemia usually coexist (1). Evidence showed that hypertriglyceridemia and visceral obesity predicted the prevalence of HBP in the Chinese population (2, 3). Hypertriglyceridemia represents the increased number of triglyceride-rich lipoproteins (TRLs) and their remnant lipoproteins (RLPs) in the circulation (4, 5). Compared with nascent TRLs, RLPs with smaller diameter contain more cholesterol (6). The atherosclerotic effect of RLPs is no less than that of low-density lipoprotein (LDL) (6). The content of cholesterol within RLPs is termed as remnant cholesterol (RC). Both cross-sectional study and prospective research showed that RC was associated with the development of HBP (7, 8). Moreover, elevated RC level can predict the risk of coronary heart disease, just like the increased level of LDL cholesterol (LDL-C) (9–11). Thus, it is essential to detect the RC level in hypertensive patients.

RC level can be calculated as total cholesterol (TC) minus LDL-C minus high-density lipoprotein cholesterol (HDL-C), using fasting or postprandial lipid profiles (12). Fasting RC levels in the general population should not exceed 0.8 mmol/L (13–15). Since 2016, postprandial detection of blood lipids has been recommended in the clinical practice (12). According to the European joint consensus statement from the European Atherosclerosis Society (EAS), postprandial RC level after a daily meal in the subjects with fasting RC <0.8 mmol/L should not exceed 0.9 mmol/L (12). However, the postprandial cutoff point of RC corresponding to fasting RC of 0.8 mmol/L in the Chinese population is still unclear. In this investigation, we compared the changes in blood lipids between hypertensive patients and their controls after a daily meal and further analyzed the optimal postprandial cutoff point of RC in Chinese subjects after a daily meal corresponding to fasting RC of 0.8 mmol/L.

## Methods

### Study Subjects

One hundred thirty-five inpatients aged 31 to 78 years, including 90 documented HBP patients (HBP group) and 45 non-HBP controls (CON group), were recruited in this study in the Department of Cardiovascular Medicine of the Second Xiangya Hospital, Central South University. All subjects were invited to fill out a questionnaire about their medical history and use of medication before participation. Inclusion criteria for the HBP group were as follows: patients with history of systolic blood pressure values ≥140 mm Hg and/or diastolic blood pressure values ≥90 mm Hg for at least 3 days (16, 17). Inclusion criteria for the CON group were as follows: contemporaneous controls without clinical history and manifestation of HBP. Exclusion criteria were secondary HBP, diabetes, thyroid diseases, liver and kidney disease dysfunction, autoimmune disease, cancer, or other severe serious medical illnesses. This study was approved by the Ethics Committee of the Second Xiangya Hospital of Central South University, and informed consent was gained from all participants.

### Specimen Collection

After at least 12 h of overnight fasting, venous blood samples were collected in all subjects before (i.e., 0 h) and at 2 h, 4 h after taking a daily breakfast according to their daily habits, such as steamed bread, rice porridge, or noodles. All subjects were required to complete the meal in 15 min. All subjects took antihypertensive drugs as usual, including angiotensin-converting enzyme inhibitor, angiotensin receptor blocker, calcium-channel blockers, diuretics, and so on. Most patients took at least two antihypertensive drugs, and blood pressure was monitored throughout the process.

### Laboratory Assays

Blood samples were analyzed as described previously (11, 18). Briefly, serum levels of TC, triglyceride (TG), and HDL-C were measured by a laboratory technician who had no knowledge of this study. LDL-C level was calculated using the Friedewald formula: LDL-C = TC − (HDL-C) − (TG/2.2) when TG was <4.5 mmol/L; otherwise, it was directly measured by chemical masking method. RC and non–HDL-C levels were estimated by the following formula: RC = TC − (HDL-C) − (LDL-C), non–HDL-C = TC − (HDL-C).

### Statistical Analysis

Quantitative variables were expressed as mean ± standard deviation for normal distribution and median and quartile for skew distribution unless specifically explained, and qualitative variables were expressed as numbers and percentages. Differences between the intragroup and intergroup means were analyzed by unpaired *t*-test or one-way analysis of variance. Categorical variables were compared using χ^2^ test. Overweight/obesity was defined as body mass index ≥24 kg/m^2^. The area under the curve (AUC) was estimated by the trapezoid method. The optimal cutoff point for postprandial RC was determined using receiver operating characteristic (ROC) curve analysis (11). All statistical analyses were performed using SPSS version 25.0, and two-tailed *P* < 0.05 was considered statistically significant.

## Results

### Clinical Characteristics and Fasting Blood Lipids of Two Groups

There was no significant difference in age, gender, body mass index, heart rate, and the percentage of current smoking between the two groups. The HBP group had a higher percentage of overweight/obesity and diastolic and systolic blood pressures than the CON group; however, the difference did not reach statistical significance. Moreover, fasting levels of TC, TG, non–HDL-C, and RC were significantly higher in the HBP group (*P* < 0.05), whereas those of HDL-C and LDL-C were similar between the two groups (Table 1).

**Table 1:** Comparison of clinical characteristics and fasting blood lipids of the two groups.

	**HBP (*n* = 90)**	**CON (*n* = 45)**
Age (y)	53 (46–62)	54 (49.5–60)
Male, *n* (%)	51 (56.67)	22 (48.89)
BMI (kg/m^2^)	23.63 (22.17–26.24)	23.46 (21.03–24.55)
Overweight, *n* (%)	37 (41.11)	12 (26.67)
Systolic pressure (mm Hg)	133.5 (120.75–149.25)	128 (121–136.5)
Diastolic pressure (mm Hg)	84 (73–92)	80 (74.5–88)
Heart rate (bpm)	78 (69.75–87)	77 (68–88)
Current smoking, *n* (%)	20 (22.22)	12 (26.67)
TC (mmol/L)	4.46 ± 0.92^*^	4.09 ± 0.66
HDL-C (mmol/L)	1.15 ± 0.29	1.20 ± 0.29
LDL-C (mmol/L)	2.56 ± 0.76	2.41 ± 0.49
TG (mmol/L)	1.48 (1.075–1.98)^*^	1.07 (0.78–1.29)
nonHDL-C (mmol/L)	3.31 ± 0.86^*^	2.89 ± 0.52
RC (mmol/L)	0.75 ± 0.37^*^	0.49 ± 0.16

*BMI, body mass index; bpm, beats/min; TC, total cholesterol; HDL-C, high-density lipoprotein cholesterol; LDL-C, low-density lipoprotein cholesterol; TG, triglyceride; non–HDL-C, non–high-density lipoprotein cholesterol; RC, remnant lipoprotein cholesterol;*

* *P < 0.05 when compared with CON group*.

### Postprandial Changes in Serum Levels of Blood Lipids in the Two Groups

**Figure 1:**
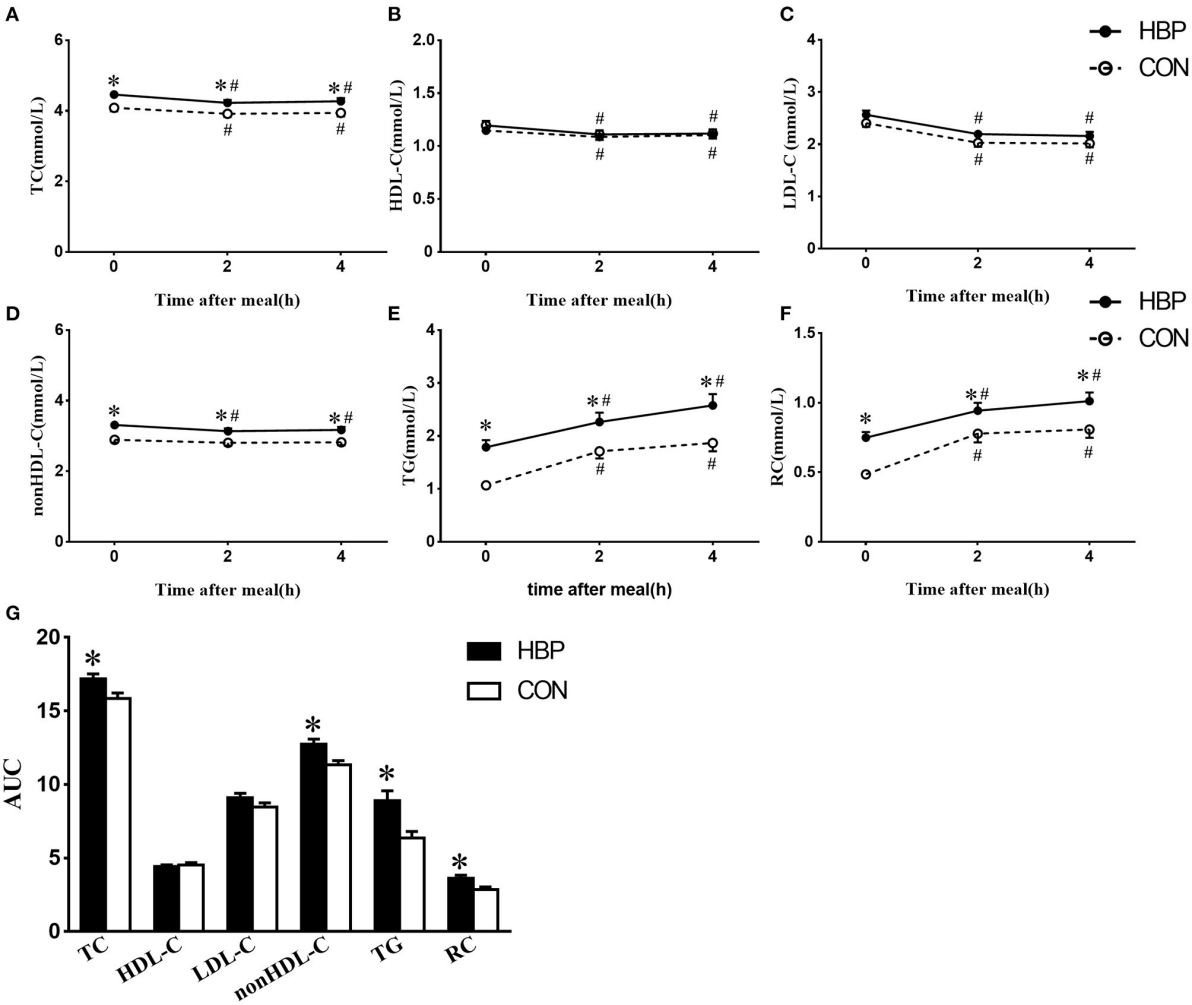
Changes in serum levels of blood lipids after a daily meal in the two groups. **(A–F)** Postprandial changes in serum levels of TC, HDL-C, non–HDL-C, LDL-C, TG, and RC after a daily meal in the HBP group (solid line) and CON group (dotted line). The bar represents standard error of the mean. **(G)** Comparison of AUC of blood lipids after a daily meal between the two groups. *P < 0.05 when compared with the CON group at the same time point. ^#^*P* < 0.05 when compared with the fasting level in the same group.

After a daily breakfast, the levels of TC, HDL-C, and LDL-C significantly reduced in the two groups (Figures 1A–C). Postprandial non–HDL-C level significantly decreased in the HBP group (*P* < 0.05) but not in the CON group (Figure 1D). Postprandial levels of TG and RC increased tremendously in the two groups (*P* < 0.05, Figures 1E,F).

Postprandial levels of TC, TG, non–HDL-C, and RC in the HBP group were significantly higher than those in the CON group, whereas there was no significant difference in the postprandial levels of HDL-C and LDL-C between the two groups (*P* < 0.05, Figures 1A–F).

AUCs of TC, TG, non–HDL-C, and RC in the HBP group were significantly higher than those in the CON group (*P* < 0.05); however, AUCs of HDL-C and LDL-C were similar in the two groups (Figure 1G).

### The Contribution of Blood Lipids to HBP

To determine the contribution of blood lipids to HBP, forward selection logistic regression analysis was performed. Among all the lipid profiles at fasting state, only the fasting RC level independently contributed to the occurrence of HBP [odds ratio (OR), 68.869; 95% confidence interval (CI), 8.533–554.560; *P* < 0.001, Supplementary Table 1].

For the close relationship between fasting and postprandial RC levels at 2 h (*r* = 0.73, *P* < 0.001) and 4 h (*r* = 0.64, *P* < 0.001), the contribution of postprandial RC levels to HBP was also evaluated by forward selection regression analysis. When age, sex, body mass index, smoking history, and RC level at each timepoint were included in covariates, postprandial RC level at 4 h also independently contributed to the occurrence of HBP (OR, 2.435; 95% CI, 1.044–5.675; *P* = 0.039; Supplementary Tables 2–4) in addition to fasting RC level.

### Determination of the Postprandial Optimal Cutoff Point Corresponding to Fasting High RC

**Figure 2:**
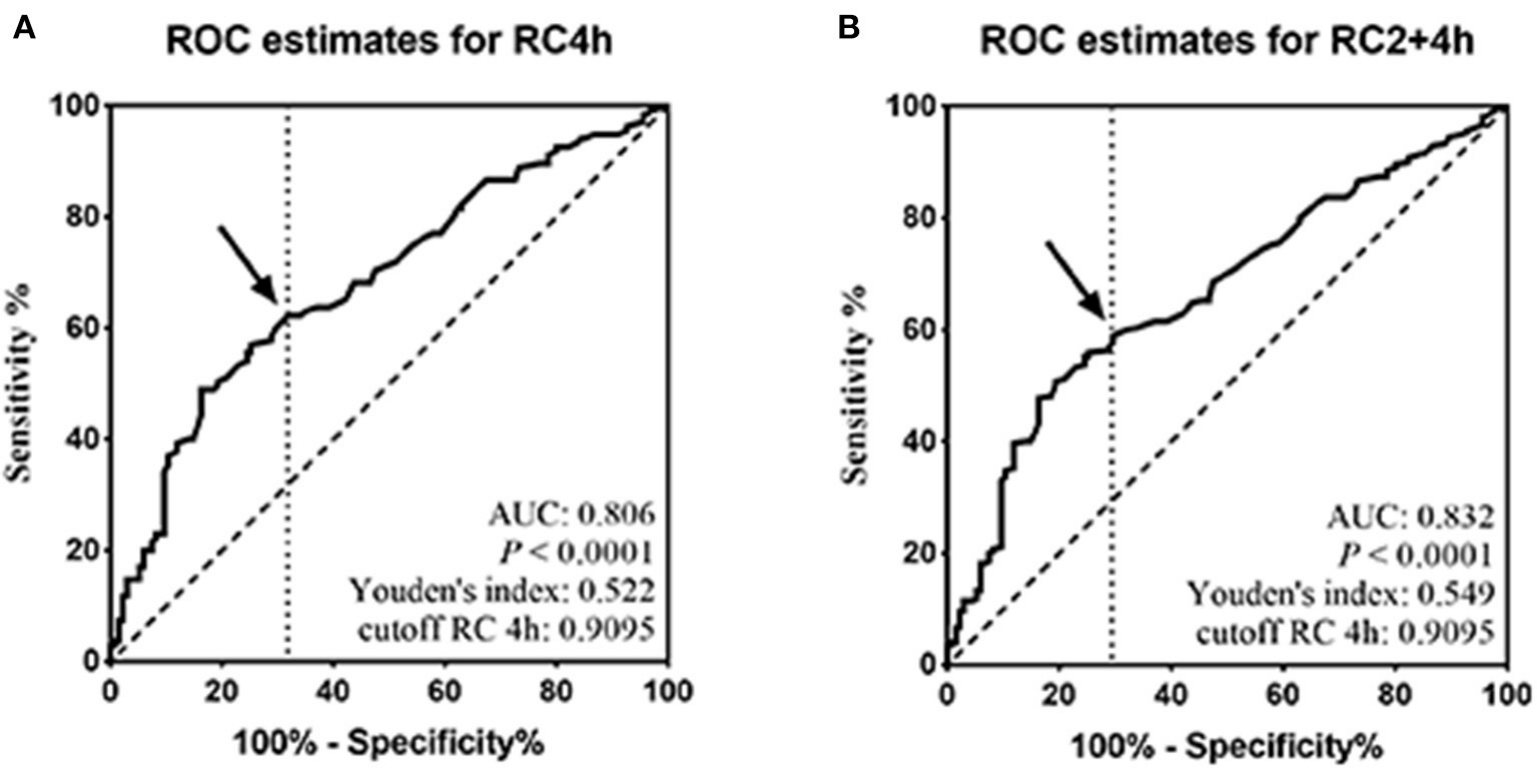
Determination of the postprandial optimal cutoff point corresponding to fasting high RC. **(A,B)** ROC analysis and Youden index determined a cutoff point for postprandial HRC at 4 h (pRC4 h) or at both 2 and 4 h (pRC2+4 h) after a daily meal; the cutoff point was indicated by the solid arrow.

Considering that the postprandial RC level reached peak value at 4 h after a daily breakfast and the independent contribution of the RC level at 4 h to HBP, ROC analysis was performed at 4 h. The optimal cutoff point for RC at 4 h to predict high RC (HRC) in relation to fasting RC of 0.8 mmol/L was 0.9095 ≈ 0.91 mmol/L (sensitivity 82.1%, specificity 70.1%, and AUC 0.806; *P* < 0.001; Figure 2A), which was close to the optimal cutoff point, 0.9 mmol/L, after a daily meal recommended by the EAS expert consensus. Moreover, when RC levels at 2 and 4 h were pooled together, ROC analysis also showed 0.9095 mmol/L as the optimal cutoff point after a daily meal (sensitivity 83.9%, specificity 71.0%, and AUC 0.832; *P* < 0.001; Figure 2B).

### Comparisons of the Percentages of Postprandial HRC Between the Two Groups

**Figure 3:**
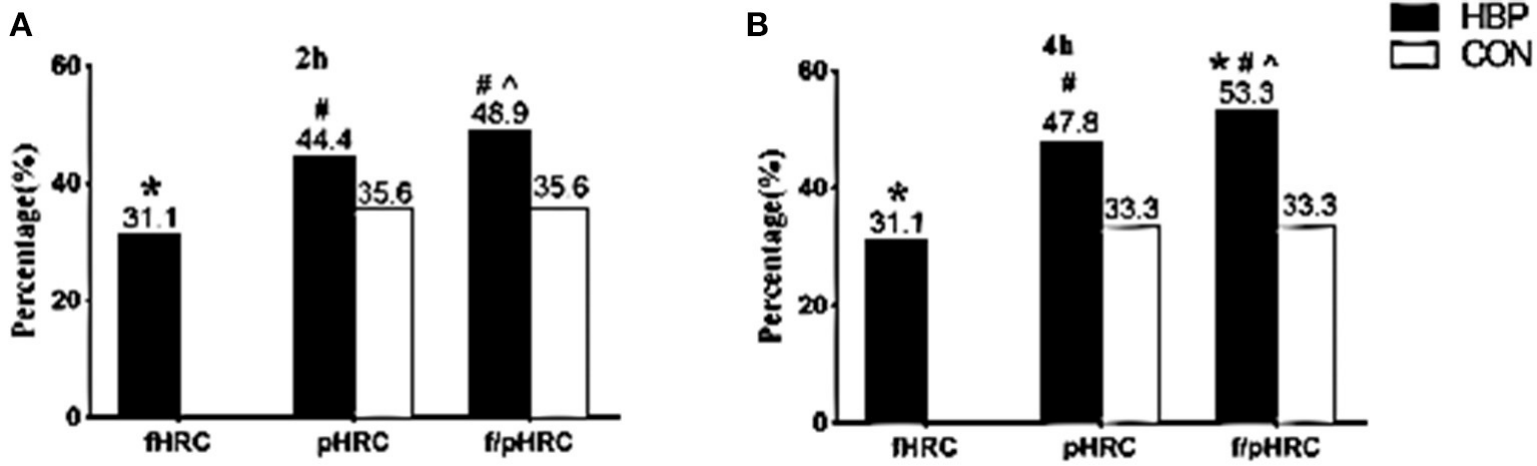
Comparisons of the percentages of HRC between the two groups at different states. **(A,B)** Comparisons of the percentages of fasting HRC only (fasting RC ≥0.8 mmol/L, fHRC), postprandial HRC only (postprandial RC ≥0.9 mmol/L, pHRC), either fasting or postprandial HRC (fasting RC ≥0.8 mmol/L or postprandial RC ≥0.9 mmol/L, f/pHRC) at 2 or 4 h after a daily breakfast. *P < 0.05 when compared with CON group. ^#^*P* < 0.05 when compared with the percentage at fasting state in the HBP group. ^∧^*P* < 0.05 when compared with the percentage of pHRC in the HBP group.

According to the optimal cutoff points of RC recommended by the EAS expert consensus (10), fasting HRC (≥0.8 mmol/L) was found in 31.1% of subjects in the HBP group; however, the percentages of postprandial HRC (≥0.9 mmol/L) significantly increased to 44.4% at 2 h and 47.8% at 4 h, respectively (*P* < 0.05). When the RC level was detected at both fasting and postprandial states at 2 or 4 h in the same subjects, the percentages of HRC significantly increased to 48.9% or 53.3% (*P* < 0.05, Figures 3A,B).

Although no subject in the CON group had fasting RC level ≥0.8 mmol/L, postprandial HRC (≥0.9 mmol/L) was found in 35.6% of subjects at 2 h and in 33.3% at 4 h in the CON group, respectively (*P* < 0.05, Figures 3A,B).

## Discussion

In this study, the optimal cutoff point of postprandial RC level after a daily meal corresponding to fasting RC level of 0.8 mmol/L was first determined in Chinese subjects. Interestingly, it was close to that recommended by the EAS expert consensus (12). Moreover, higher RC level and higher proportion of HRC were found in the HBP group in both fasting and postprandial states, suggesting that HBP patients could be at greater cardiovascular risk due to abnormal TRL metabolism, in addition to HBP.

RC level can be accurately detected through several expensive and complex methods, including ultracentrifugation, nuclear magnetic resonance, immune separation, and so on (19–21). However, those kinds of accurate detection are very difficult to be widely used in the primary hospitals. The formula method recommended by the EAS expert consensus gives doctors the opportunity to estimate the RC level without additional cost (12). Elevation of the RC level indicated the excessive overproduction of nascent TRLs and/or delayed removal of RLPs in patients with HBP. And this situation persisted in the postprandial state. Similar conditions were also found in the TG level. Those results suggested that there is a close relationship between HBP and abnormal metabolism of TRLs/RLPs.

There are several mechanisms that can explain the relationship between TRLs/RLPs and the development of HBP. First, TRLs and RLPs could directly and indirectly promote the production of aldosterone, which plays an important role in the pathogenesis of HBP (22–24). It was shown that the expression and secretion of aldosterone in adrenal cells were induced by very-low-density lipoproteins, the main ingredients of TRLs (25–27). One possible explanation is that phospholipase D mediated aldosterone synthase expression (27). Second, RLPs can cause endothelial dysfunction, which is one of the key mechanisms of HBP (28). Postprandially increased RLPs can directly impair arterial vasodilation of the separated vascular ring through inducing oxidative damage (29). In addition, clinical studies also showed the relationship between RC and HBP. It was found that high fasting RC level was associated with the development of HBP after 10 years in Japanese subjects who had normal blood pressure at baseline (8). Winkler et al. (7) also reported that TRLs were associated with HBP in preeclampsia. These findings indirectly support the potential contributions of HTG to HBP.

It is well-known that people spend most of the day at the postprandial state. Moreover, a considerable number of subjects visit the medical services at the postprandial state. Thus, some patients with high TG or RC level may be missed if blood lipids were limited to detect at the fasting state. According to the EAS expert consensus and the statement from the American Heart Association, the postprandial TG level in an individual with fasting TG <1.7 mmol/L should not increase above 2.0 and 2.26 mmol/L, respectively, after consuming a daily meal (12, 15). Recently, we determined a cutoff point for postprandial TG level 2.02 mmol/L at 4 h after a daily breakfast corresponding to fasting TG level 1.7 mmol/L in Chinese subjects (30), which is close to the cutoff point for postprandial TG level 2.0 mmol/L recommended by the EAS expert consensus (12). However, as expected of the EAS expert consensus, there was no recommendation about fasting and postprandial RC levels in the United States or China.

In this study, the cutoff point of postprandial RC level corresponding to fasting RC level of 0.8 mmol/L was ~0.91 mmol/L after a daily meal in Chinese subjects, which was quite near to that recommended by the EAS expert consensus, i.e., 0.9 mmol/L (12). It suggests that Chinese subjects may share similar cutoff points with the Europeans after a daily meal. When postprandial RC of 0.9 mmol/L was used to evaluate the percentage of postprandial HRC in each group, postprandial HRC was found in more hypertensive patients and about one-third controls. The postprandial increase in HRC in the two groups could be associated not only with their own habitual breakfasts, but also with the existence of some subjects with overweight/obesity and smoking in each group. Decreased hydrolysis of TRLs was reported in smokers and patients with obesity (31–33). If both fasting and postprandial blood lipids were detected in a certain subject, the diagnostic rate of HRC could be further improved, although it is not feasible in the real world. Certainly, the detection of postprandial RC level can find more patients with HRC and is worth carrying out in the clinical practice.

This study is associated with several limitations. First, the number of cases in this study was relatively small compared to other similar studies (34). Second, 94% of subjects had breakfasts in the hospital canteen, which could be different from their usual diets at home, although they can freely choose food according to their daily habits. Third, LDL-C and RC levels were calculated by Friedewald formula, which may cause deviation with those directly measured or calculated by other formulas (35).

In conclusion, postprandial RC level increased significantly after a daily meal, and hypertensive patients had significantly higher percentage of HRC than the controls. More importantly, the postprandial detection of blood lipids could be helpful to find HRC.

## Data Availability Statement

The raw data supporting the conclusions of this article will be made available by the authors, without undue reservation.

## Ethics Statement

The studies involving human participants were reviewed and approved by Ethics Committee of the Second Xiangya Hospital of Central South University. The patients/participants provided their written informed consent to participate in this study.

## Author Contributions

JX, PQ, QX, LG, LZ, YT, and YF carried out the experimental work and the data collection and interpretation. PQ, XD, TW, and LL participated in the design and coordination of experimental work. JX, PQ, and LL carried out the study design, the analysis and interpretation of data, and drafted the manuscript. All authors contributed to the article and approved the submitted version.

## Conflict of Interest

The authors declare that the research was conducted in the absence of any commercial or financial relationships that could be construed as a potential conflict of interest.

## Abbreviations

AUC: area under the curve
CON: non-HBP controls
EAS: European atherosclerosis society
HBP: hypertension
HDL-C: high-density lipoprotein cholesterol
HRC: high remnant cholesterol
LDL: low-density lipoprotein
LDL-C: low-density lipoprotein cholesterol
non–HDL-C: non–high-density lipoprotein cholesterol
RC: remnant cholesterol
RLPs: remnant lipoproteins
ROC curve: receiver operating characteristic
TC: total cholesterol
TG: triglyceride
TRLs: triglyceride-rich lipoproteins.
