# Supplementary material for: Change in Postprandial Level of Remnant Cholesterol After a Daily Breakfast in Chinese Patients With Hypertension
Source: Front Cardiovasc Med. 2021 Jun 15;8:685385. doi: 10.3389/fcvm.2021.685385 (PMC8239280; doi:10.3389/fcvm.2021.685385)
Supplement: Supplementary file 1 [file Data_Sheet_1.docx]

Supplementary data

Table S1: Odds ratios and scores of fasting plasma lipid levels for development of hypertension.

| Factor | OR/Score | *P* value |
| --- | --- | --- |
| TG | 0.197 | 0.999 |
| TC | 0.680 | 0.764 |
| HDL-C | 0.83 | 0.563 |
| LDL-C | 0.361 | 0.548 |
| nonHDL-C | 0.361 | 0.548 |
| **RC** | **4.232** | **<0.001** |

Table S2: Odds ratios and scores of fasting plasma RC level and population parameters for development of hypertension.

| Factor | OR/Score | *P* value |
| --- | --- | --- |
| Age | 0.101 | 0.751 |
| Gender | 0.782 | 0.377 |
| Smoker | 0.323 | 0.570 |
| BMI | 0.694 | 0.405 |
| **RC 0 h** | **4.232** | **<0.001** |

Table S3: Odds ratios and scores of plasma RC level at 2 h after a daily meal and population parameters for development of hypertension.

| Factor | OR/Score | *P* value |
| --- | --- | --- |
| Age | 0.340 | 0.560 |
| Gender | 0.731 | 0.393 |
| Smoker | 0.722 | 0.396 |
| BMI | 3.612 | 0.057 |
| RC 2 h | 3.175 | 0.075 |

Table S4: Odds ratios and scores of plasma RC level at 4 h after a daily meal and population parameters for development of hypertension.

| Factor | OR/Score | *P* value |
| --- | --- | --- |
| Age | 0.097 | 0.756 |
| Gender | 0.730 | 0.393 |
| Smoker | 0.396 | 0.529 |
| BMI | 2.361 | 0.124 |
| **RC 4 h** | **2.435** | **0.039** |

Scores, mean the changes of the residual of the regression model when insignificant covariates were included in the equation compulsorily.
